# Treatment of Open Traumatic Medial Malleolus Bone Loss With Osteochondral Allograft: A Case Report

**DOI:** 10.7759/cureus.31755

**Published:** 2022-11-21

**Authors:** Thomas B Anderson, Andrew S Bae, Jazmone Kelly, David P Antekeier

**Affiliations:** 1 Orthopaedic Surgery Residency, Jack Hughston Memorial Hospital, Phenix City, USA; 2 Osteopathic Medicine, Philadelphia College of Osteopathic Medicine, South Georgia, Moultrie, USA; 3 Orthopaedic Surgery, Hughston Clinic, Columbus, USA

**Keywords:** type iiia open medial malleolus fracture, bone loss, ankle arthritis, ankle trauma, medial malleolus, fresh osteochondral allograft

## Abstract

Osteochondral damage to the ankle joint can be a difficult problem to manage in a young active patient. There are several described surgical treatments ranging from cartilage repair techniques to arthrodesis and ankle replacement. In this case, we present a 28-year-old male who sustained a right type IIIA open medial malleolus fracture following an all-terrain vehicle crash. After sharp debridement, the clinical decision was made to treat the patient with an osteochondral allograft. At one- and two-year post-allograft reconstruction, radiographs demonstrated good incorporation of the graft. The patient was ambulating with no pain or assistive devices. Our case report specifically describes the successful treatment of a traumatic medial malleolus ankle fracture with bone loss using an osteochondral allograft in a young active patient.

## Introduction

Osteochondral damage to the ankle joint can be a difficult problem to manage in a young active patient. There are described surgical treatments that range from cartilage repair techniques to arthrodesis and ankle replacement. Arthrodesis may provide relief of pain but can become problematic causing functional limitations in an active patient.

The use of osteochondral allograft is not a novel technique as it has been used in the setting of malignant bone tumors [1]. However, there is no documented literature on medial malleolus osteochondral allograft in the setting of traumatic medial malleolus bone loss. Cases of autologous bone grafts have been reported as well as a recent report on distal tibia bone sliding. Neither option offers a good solution to restore the articular surface, especially for a young patient. We present a viable option with good outcomes for a young patient after he sustained a traumatic medial malleolus ankle fracture with significant bone loss.

## Case presentation

**Figure 1:**
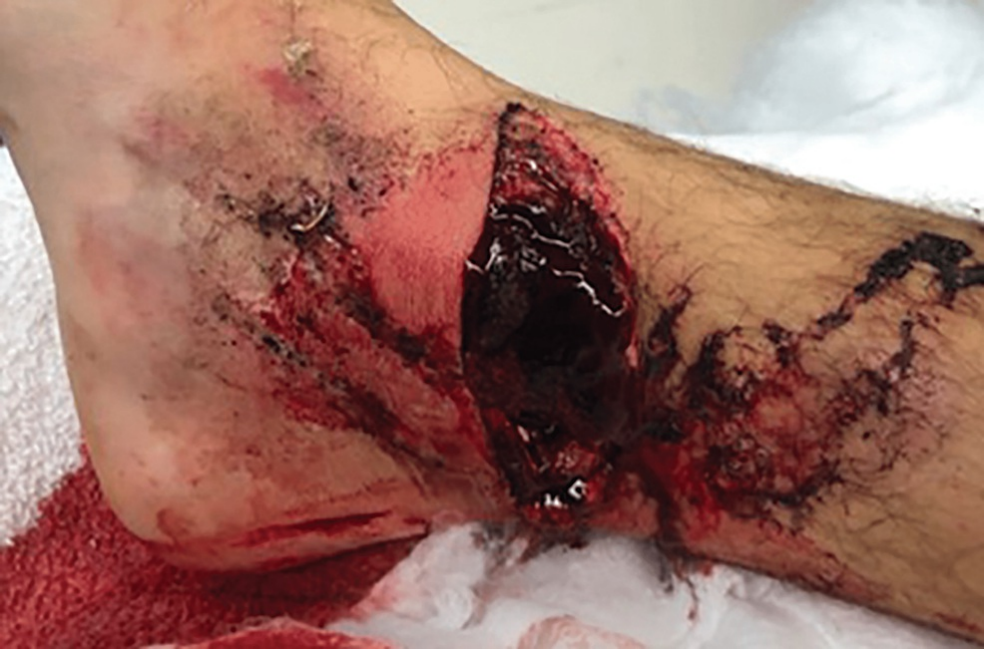
Right medial ankle wound with gross contamination.

**Figure 2:**
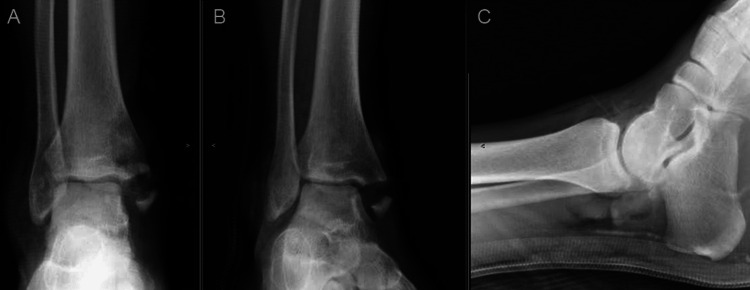
Initial injury radiographs - AP, oblique, and lateral of ankle AP: anteroposterior

A 28-year-old male sustained an open injury to his right ankle while riding an all-terrain vehicle (ATV) on the evening of October 5, 2019. The patient recalls that the injury was a result of the ATV rolling over while accelerating between 15 and 20 miles per hour (mph). His right foot was caught under the ATV as the vehicle flipped. He had an immediate onset of pain, deformity, and inability to bear weight. He was brought to the emergency department for an initial assessment. A full examination of the ankle was not possible due to significant hemarthrosis. A large transverse laceration was noted over the medial ankle with gross contamination (Figure 1). Plain radiographs of the right ankle revealed a comminuted displaced medial malleolus fracture (Figure 2). He proceeded to the operating room the same night for incision and debridement of the open fracture and wound.

**Figure 3:**
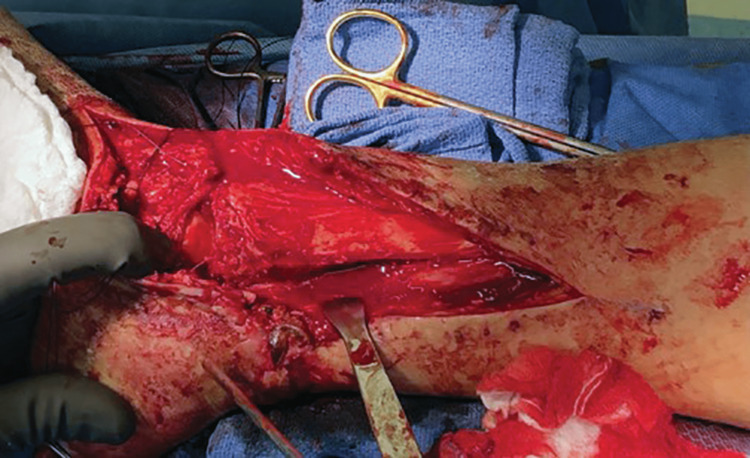
Intraoperative media of medial malleolus bone loss.

The patient was placed supine and draped in a sterile manner. Examination showed a 10-cm transverse incision extending from the anteromedial to the posteromedial aspect of his right ankle. Also, there was a segmental portion missing from the anterior shoulder to the posterior colliculus which extended for approximately 3 x 1.5 cm (Figure 3). The deltoid origin was still intact and there was a very small fragment of bone present distally. Examination showed that the deep peroneal nerve was intact. There was a complete transection of the posterior tibial tendon, avulsion of the flexor digitorum longus muscle belly, and the posterior tibial artery was avulsed and occluded. There was an intact posterior tibial vein, posterior tibial nerve, flexor hallucis longus, and Achilles tendon. We found grass, dirt, and gravel in the wound. Sharp excision was used to remove devitalized skin, subcutaneous tissue, muscle, and bone. Following the debridement, the wound was thoroughly irrigated with 9 L of normal saline. Next, the posterior tibial tendon ends were tagged with a PDS II (polydioxanone) suture. In addition, the flexor digitorum tendon was preserved for a tenodesis during the reconstructive phase. The patient was taken back to the operating room for a total of three debridement procedures with splinting performed following each debridement. Following initial debridement, a peroneal nerve palsy was identified. A working diagnosis of type IIIA open medial malleolus fracture was made, and the patient was scheduled for open reduction internal fixation surgery with allograft reconstruction on November 13, 2019.

**Figure 4:**
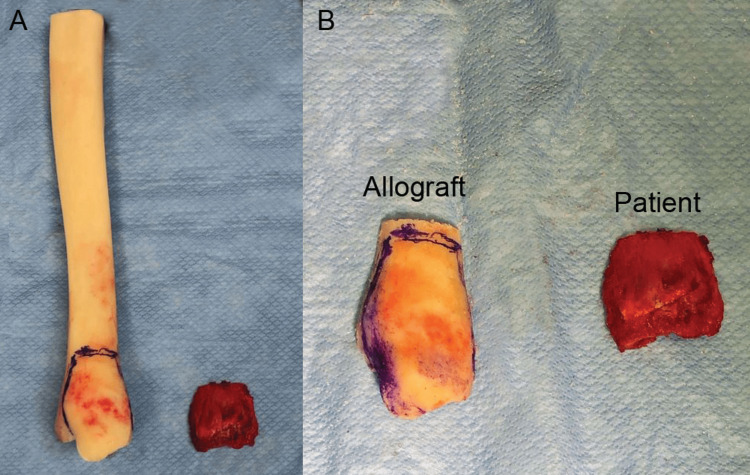
Osteochondral tibia allograft sizing.

**Figure 5:**
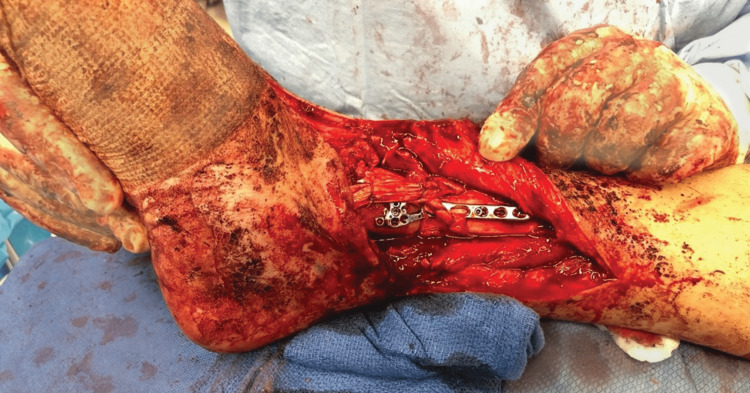
Medial malleolus osteochondral allograft with overlying hardware and tibialis anterior tendon allograft reconstruction.

The patient was taken back to the operating room, placed supine with a hip bump, and draped in a sterile fashion. A standard medial malleolus approach was used to access the defect. An anatomically aligned fresh osteochondral tibia allograft was sized for placement over the defect (Figure 4). It was then placed in the defect and temporarily fixated with 2.0 Kirschner wires. The graft was secured with two 2.7-mm screws that were placed using a lag screw technique. A distal radius plate was placed and bent to the shape of the allograft to provide stabilization and buttress to the medial joint line. Reconstruction of the medial complex was then performed with an anterior tibialis allograft. This was placed through the deltoid ligament around the allograft and remained extra-articular. Using a suture anchor, this was then stabilized to the tibia. Tensioning was performed of the deltoid and then an additional suture anchor was placed tensioning the deltoid/anterior tibialis allograft (Figure 5). An additional suture anchor was placed anteriorly. At the same time, the anterior capsule was applied to the native tissue and to the allograft bone. In addition, a posterior suture anchor was placed stabilizing the posterior structures to the native bone. The ankle was found to be stable and bone marrow aspirate from the iliac crest was added to the allograft. Upon closing, there was a notable 1.5 cm skin defect for which we used Integra to help close the gap. A wound vac was then applied. Intraoperative radiographs demonstrated good placement of the osteochondral allograft. The patient was placed into a short leg splint with stirrups and made non-weight bearing on the right lower extremity.

**Figure 6:**
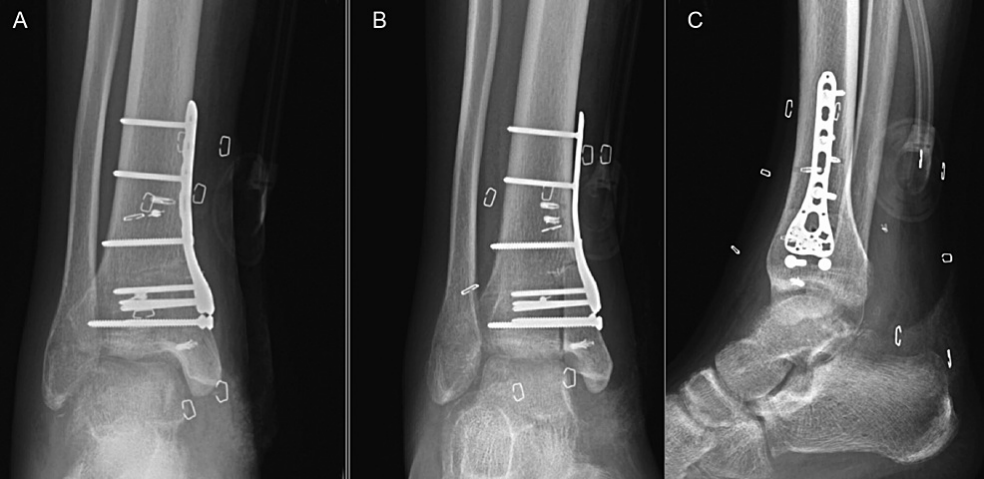
Radiographs of the right ankle two weeks status post-allograft implantation with no interval displacement.

At two weeks postoperatively, he was doing excellent. His pain was well controlled, and he had no complaints. His gross motor and sensory examination remained intact except for peroneal nerve palsy and mild foot drop on the right. Three views of radiographs of the right ankle demonstrated intact allograft and hardware with no interval displacement (Figure 6). Sutures were removed at this time, and he was instructed to begin range of motion exercises of the ankle and digits. He was placed into a controlled ankle motion (CAM) walker boot and told to remain non-weight bearing on the right lower extremity for another six weeks.

**Figure 7:**
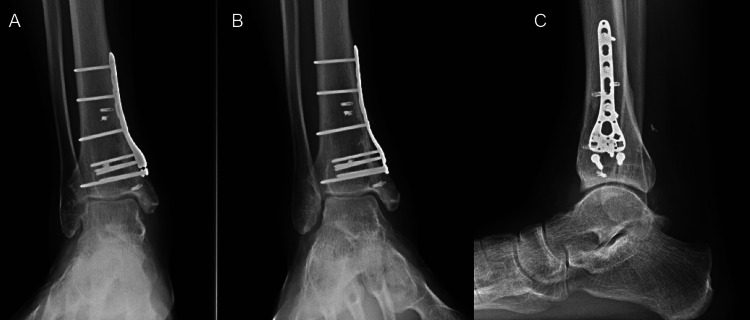
Radiographs of right ankle at year two post-allograft implant with good incorporation of graft.

When the patient presented at six-week postoperatively, he had no changes in his examination or new complaints. At one-year status post-allograft reconstruction, he was able to ambulate independently. He denies instability in the ankle. There was no fever or any sign of infection. Symptoms from the right peroneal palsy were improved. At two-year post-allograft reconstruction, he presented once more with no pain with ambulation or any other complaints. His exam showed an equal dorsiflexion range of motion to the contralateral side. Repeat radiographs of the right ankle demonstrated intact implants with significant callus formation, incorporation of the allograft, and no signs of osteoarthrosis (Figure 7).

## Discussion

Ankle fractures are among the most common fractures with an incidence of 18.7 per 100,000 individuals and accounting for 9% of all fractures managed by orthopedic surgeons [2]. Isolated malleolus fractures occur 70% of the time. Open ankle fractures are relatively rare, occurring only 2% of the time [3]. The stability of the ankle joint relies heavily on the malleoli and ligamentous structures. Typically, open isolated medial malleolus fractures are treated with initial sharp excisional debridement and washout followed by an immediate or staged open reduction internal fixation [4]. Depending on the amount of bone loss, a trial of conservative management in a cast with non-weight bearing can be effective. Primary arthrodesis of the ankle joint is an option, depending on the age and functional status of the patient. However, this decision should be weighed heavily as there are functional restrictions associated with joint fusion. Surgeons have received favorable results using several techniques for open medial malleolus fractures with bone loss.

Huang et al. reported on the use of a bone sliding technique as a reconstruction option in the setting of traumatic medial malleolus bone loss in a middle-aged diabetic female [5]. In his report, Huang describes cutting a small portion of the tibia just proximal to the defect and sliding it distally in place of the medial malleolus. The use of periosteum in the articular surface could potentially produce cartilage in the setting of stress. Results at six months postoperatively showed consolidation of the sliding bone block. The patient was satisfied with the outcome despite having stiffness.

There are reports of primary reconstruction for medial malleolus bone loss with open reduction internal fixation, fibular head free flap graft, and iliac crest autograft [6,7]. Patients who undergo primary open reduction internal fixation demonstrated more chronic ankle swelling postoperatively [8]. Lughi et al. report the use of a biologic decellularized human dermal matrix in their case report that provided a stable ankle joint at short-term follow-up [9]. There are several reports of medial malleolus reconstruction with iliac crest bone graft that show favorable results [8,10]. However, there were some restrictions noted in a range of motion at follow-up. Nithyananth et al. describe a 25-year-old patient who sustained a crush injury with medial malleolus bone loss [11]. In the report, he described reconstruction with autologous iliac crest bone graft produced a stable and functional ankle joint. The potential downside to this technique is the ankle is a weightbearing joint and replacing the cartilaginous bone with cortical bone can lead to post-traumatic arthrosis.

Our report describes the successful incorporation of osteochondral allograft for medial malleolus bone loss. This technique is extremely beneficial in the setting of a young patient as the defect over the articular surface is replaced with cartilage. In addition to providing joint stability, restoration of the articular surface will in theory reduce post-traumatic arthrosis. Short-term follow-up with other techniques demonstrates favorable results except for the loss of dorsiflexion or plantarflexion. Retained range of motion and stability of the ankle postoperatively is in part due to the reconstruction of the deltoid and anterior tibialis tendon. Regardless of the technique used to restore the medial aspect of the ankle, patients often demonstrate some osteoarthrosis at the two-year follow-up. Interestingly, our case demonstrates excellent ankle joint space at a two-year follow-up with no signs of osteoarthrosis and no pain with ambulation.

## Conclusions

Our case describes a young patient with significant bone loss over the medial malleolus and gross contamination in a traumatic setting. Prior reports describe bone sliding, temporary placement of antibiotic beads, fusion using an anterior plate, fibular head graft, and iliac crest bone graft. Our case demonstrates the utilization of osteochondral allograft to be a viable option. At two-year postoperatively, our patient had no pain, full range of motion, and an excellent outcome. Osteochondral tibia allograft is a viable long-term option in young patients with medial malleolus bone loss that offers pain relief, preserves joint function, and reduces ankle arthrosis.
